# Correlation and comparison of quadriceps endurance and knee joint position sense in individuals with and without unilateral knee osteoarthritis

**DOI:** 10.1186/s12891-022-05403-9

**Published:** 2022-05-12

**Authors:** Mastour Saeed Alshahrani, Ravi Shankar Reddy, Faisal Asiri, Jaya Shanker Tedla, Adel Alshahrani, Praveen Kumar Kandakurti, Venkata Nagaraj Kakaraparthi

**Affiliations:** 1 Department of Medical Rehabilitation Sciences, College of Applied Medical Sciences, King Khalid University, Abha, Saudi Arabia; 2 Department of Physical Therapy, College of Applied Medical Sciences, Najran University, Najran, Saudi Arabia; 3 College of Health Sciences, Gulf Medical University, Ajman, United Arab Emirates

**Keywords:** Endurance, Proprioception, Position sense, Osteoarthritis

## Abstract

**Background:**

Knee osteoarthritis (KOA) is a painful degenerative joint disease that may limit activities of daily living. This study aimed to determine the relationship between quadriceps endurance and knee joint position sense (JPS) in KOA individuals and compare the quadriceps endurance and knee JPS with and without KOA.

**Methods:**

This comparative cross-sectional study was conducted in medical rehabilitation clinics, King Khalid University, Saudi Arabia. This study recruited 50 individuals diagnosed with unilateral KOA (mean age = 67.10 ± 4.36 years) and 50 asymptomatic individuals (mean age = 66.50 ± 3.63 years). Quadriceps isometric endurance capacity (sec) was measured using a fatigue resistance test, and knee JPS (degrees) were assessed using a digital inclinometer and evaluated in sitting and standing positions.

**Results:**

Quadriceps isometric endurance showed a significant moderate negative correlation with knee JPS in 20° of flexion (*r* = -0.48, *p* < 0.001); 40° of flexion: *r* = -0.62, *p* < 0.001; 60° of flexion: *r* = -0.58, *p* < 0.001) in sitting and 20° of flexion (*r* = -0.25, *p* = 0.084) in standing position in KOA individuals. When compared to the asymptomatic, the quadriceps endurance was lower (*p* < 0.001), and knee joint position errors were larger (*p* < 0.001) in KOA individuals.

**Conclusion:**

Results of this study showed that quadriceps endurance capacity is negatively associated with knee JPS. KOA individuals demonstrated lower quadriceps endurance and larger JPS compared to asymptomatic.

## Introduction

Knee osteoarthritis (KOA) is a degenerative joint disease and the most common cause of impairment and reduced mobility in the elderly, with substantial individual discomfort and high direct and indirect illness-related costs [1]. Due to a drop in hormone levels, women have a higher incidence than males following menopause [2]. KOA symptoms include pain, joint swelling, morning stiffness, decreased physical activity, limited range of motion, and limited participation in social activities [3].

Compared with healthy populations, individuals with KOA have substantially impaired proprioception [4]. Muscle weakening, joint degeneration, and reduced mechanoreceptor responsiveness may impair proprioception [5–16]. The poor proprioception of KOA subjects is a significant risk for the disease's development and maintenance [5, 11, 17]. Few researchers state that disrupted proprioceptive afferent information may be critical in pain development and tissue damage in degenerative joint disease [18]. Therefore, assessing knee joint proprioceptive sensibility and its associations with muscle endurance is essential in evaluating and managing KOA individuals.

The quadriceps muscles maintain joint stability with the hamstring muscles during lower extremity static and dynamic activities [19]. Subjects with KOA demonstrated lower knee muscle strength and endurance than healthy individuals [20]. Muscle fatigue has been shown to impair joint position sense [8, 21]. Decreased muscle strength, endurance, and fatigability are reported in subjects with KOA [22]; these changes may decline proprioceptive sensibility and motor control in these individuals. In knee dysfunctions, reposition sense has been demonstrated to improve with knee endurance exercises [23, 24]. These findings imply that quadriceps muscles are critical for preserving and enhancing knee joint position sense in people with KOA.

There are limited studies on the relationship between quadriceps endurance and proprioception in people with KOA. Quadriceps muscle endurance has been shown to be associated with the severity of the KOA [25]. Reduced muscle endurance may reduce physical activity, resulting in a reduction in total physical fitness [26]. It has been suggested that the relationship between muscle endurance and proprioceptive sensibility are essential factors in maintaining optimal motor control of persons with KOA (17). As a result, it's crucial to know whether decreased muscle endurance is linked to more significant proprioception errors at the knee, as these changes could impede people's capacity to carry out their everyday tasks. So, is the need for this study. This study aims to 1) examine the relationship between quadriceps muscle endurance capability and knee JPS in unilateral KOA individuals and 2) compare quadriceps endurance and joint position sense in unilateral KOA individuals to asymptomatic controls. We hypothesize that there may be a significant correlation between quadriceps muscle endurance holding capacity and knee JPS in individuals with unilateral KOA. Also, we expect quadriceps endurance capacity to be reduced and knee JPS errors to be more prominent in unilateral KOA individuals than asymptomatic.

## Methods

### Design and sample

This comparative cross-sectional study involving 100 individuals (50 unilateral KOA and 50 asymptomatic subjects) was carried out at King Khalid University's Medical Rehabilitation Department. These research individuals with a diagnosis of KOA were evaluated by an orthopedist or general practitioner and then referred to physical therapy for inclusion in the study. The unilateral KOA subjects included in the study were based on X-ray imaging, physical examination, and Kellgren-Lawrence (KL) grading, greater than or equal to grade 1 out of the possible four grades [27], and have clinical symptoms. Subjects were excluded; if the individuals had undergone knee surgery in the last six months; had muscle, joint injuries that limit lower limb function, rheumatic disease or cardiovascular disease, or other neurological problems. Asymptomatic individuals were included if they generally present with good health and no history of knee pain, no use of morphine, corticosteroids, or pain killers.

Before enrolling in this study, all study individuals were educated about the experiment's purpose and asked to sign an informed consent form. This research was carried out in line with the Declaration of Helsinki. The research ethics committee board at King Khalid University reviewed and approved this work (ECM#2021–4504).

### Outcome measures

#### Quadriceps isometric endurance

**Fig. 1:**
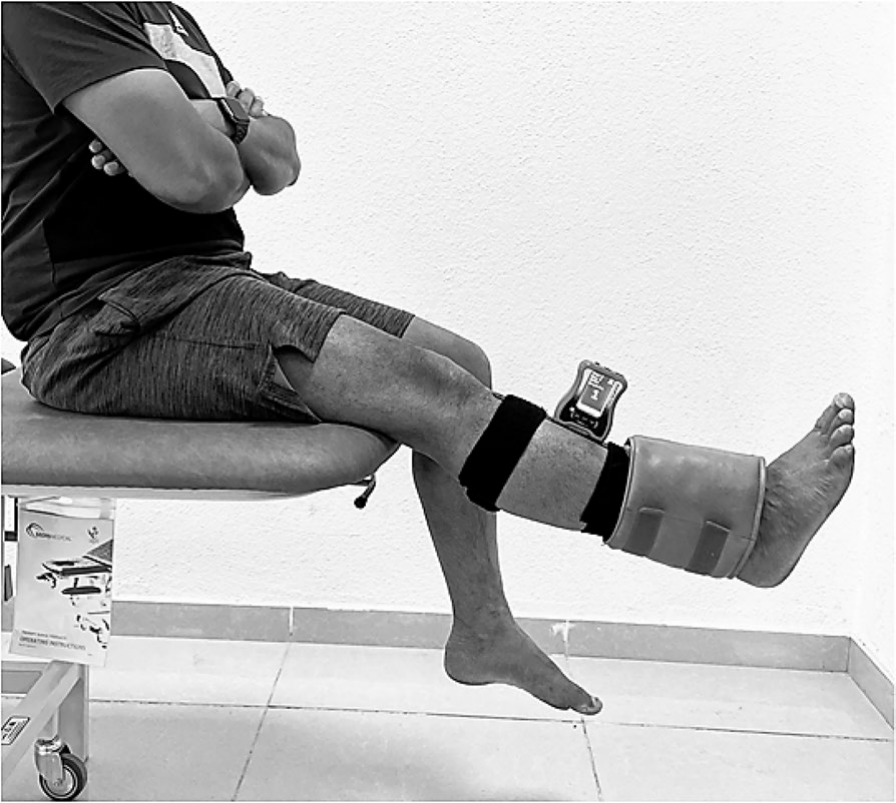
Quadriceps isometric endurance testing using a fatigue resistance test

Quadriceps isometric endurance was measured using a fatigue resistance test. The individuals sat on a chair with their back straight and their hips and knees flexed at 90 degrees. A 5 kg weight was secured with tape above the ankle (testing side), and a digital inclinometer device was attached to the shin to see the leg alignment in relation to the horizontal plane (Fig. 1).

To begin the endurance test, participants were instructed to fully extend their knees and maintain complete knee extension (horizontal position). Individuals were instructed to maintain this test position for as long as possible. A stopwatch was used to record the time. The duration of time spent in this position is used to determine their knee extensor endurance capacity. If the knee extension position shifted at least five degrees from the horizontal plane (as determined by an inclinometer), or the participant could not sustain the test leg in a horizontal position due to pain or fatigue, the test was terminated. This Endurance testing protocol was repeated three times, and the best effort was taken for analysis. There was a rest period of three minutes between each testing trial. The same investigator recorded the individuals' readings, and no additional feedback was given to the individuals during the testing time.

#### Knee joint position sense

**Fig. 2:**
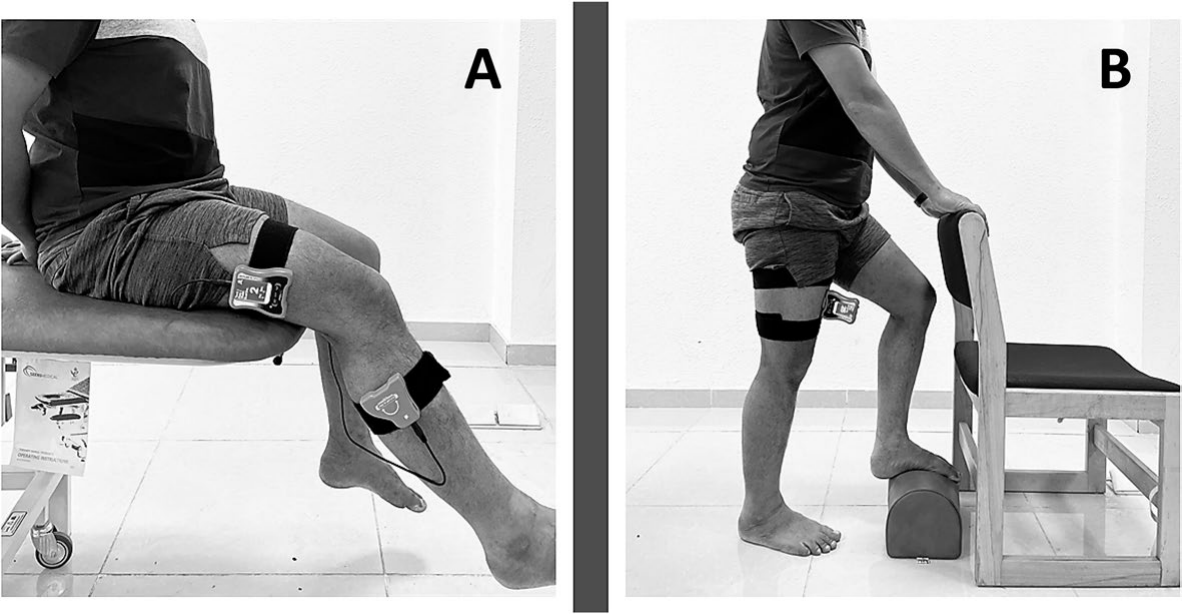
**A** Knee joint position sense (JPS) testing in sitting position: **B** knee JPS testing in standing position

We used a digital inclinometer (J-Tech Medical, Midvale, UT, USA) to measure the knee joint position sense (Fig. 2). To standardize knee proprioceptive testing, all individuals wore comfortable shoes and shorts during the testing. In addition, all individuals wore eye masks during the measurements to avoid sensory feedback. The knee JPS was tested in 20, 40, and 60 degrees in sitting and 20 degrees in standing positions.

Knee JPS in sitting position (Fig. 2A) were measured while subjects sat on a chair, and one portion of the inclinometer was attached with a strap to the lower one-third segment of the femur's lateral surface along the joint line. We attached the other portion of the inclinometer along the joint line to one-third of the lower leg's lateral segment. The examiner guided the individuals from the starting position (knee flexion of 90°) until the knee reached the target angle (20 or 40 or 60 degrees) and maintained this position for five seconds. The subjects were asked to recollect this target position, and then the knee was guided to the starting position. The subjects were then asked to reposition the leg to a target position, which they initially remembered, and the reposition accuracy was assessed in degrees.

To measure knee JPS in a standing position (Fig. 2B), the subjects adopted a standing position with a chair placed in front of the individuals. The testing leg is maintained straight with the inclinometer attached in front of the thigh and secured using a Velcro strap in mid-position. The non-testing leg was placed in front of the testing leg and rested over a firm foam step. To keep their balance while squatting with a single leg, we allowed individuals to grab the chair with two hands. The subjects were asked to squat on the testing leg until the knee angle reached 20 degrees (from complete knee extension) and asked the participant to stop for five seconds, memorize this position, and return to the starting position (to complete knee extension). The individuals were then asked to replicate the target angle as accurately as possible, and the reposition accuracy was measured. We reported the difference between the angle sensed by the individuals and their replicated angle as the absolute angular error. We took three measurements and used an average of three values for analysis.

### Statistical analysis

The Kolmogorov–Smirnov test analyzed if the study variables followed a normal distribution. Pearson correlation coefficient (r) was used to see the relationship between quadriceps endurance and knee JPS in KOA individuals. An independent t-test was used to see between-group differences in quadriceps endurance holding time and JPS of KOA and asymptomatic groups. Cohen's d is an estimate of effect size, which measures the standardized difference between two means after dividing the result by the pooled standard deviation [28]. The Statistical Package for the Social Sciences (SPSS) software (IBM, Chicago, IL, USA, version 24) was used to analyze the data of this cross-sectional study). A *p*-value of ≤ 0.05 was considered statistically significant to the study findings.

## Results

A total of 100 individuals (50 with unilateral KOA and 50 asymptomatic) were enrolled in this study, and the participant's demographic characteristics are presented in Table 1. There were no significant variations in age (years), height (cm), weight (kg), or BMI across groups (all p > 0.05).

**Table 1:** Demographic characteristics of study individuals

Variable	Knee OA individuals (*n* = 50)	Asymptomatic (*n* = 50)	*p*-value
Age (years)	67.10 ± 4.36	66.50 ± 3.63	0.457
Height (cm)	167.72 ± 11.67	170.08 ± 10.72	0.295
Weight (kg)	82.40 ± 8.89	83.46 ± 9.53	0.566
BMI (kg/m^2^)	29.59 ± 4.52	28.99 ± 3.72	0.470
VAS—Pain	4.22 ± 0.98	-	N/A
WOMAC			
• Pain	8.60 ± 4.33	-	N/A
• Stiffness	3.22 ± 3.25	-	N/A
• Physical function	38.40 ± 15.10	-	N/A

Results show as mean ± SD. *Knee OA* Knee osteoarthritis, *BMI* body mass index, *VAS* Visual analog scale, *WOMAC* Western Ontario and McMaster Universities Osteoarthritis Index, *N/A* not applicable

The association between quadriceps endurance and knee proprioceptive JPS measurements is shown in Table 2. To our expectation, quadriceps endurance showed a moderate negative correlation with JPS in 20° of flexion (*r* = -0.48, *p* < 0.001), 40° of flexion (*r* = -0.62, *p* < 0.001) 60° of flexion (*r* = -0.58, p < 0.001) in sitting positions. Furthermore, there was a moderate negative correlation with JPS in 20° of flexion (*r* = -0.25, *p* = 0.084) in the standing position.

**Table 2:** Relationship between quadriceps endurance and knee JPS in KOA (*n* = 50)

Correlate Variables	Quadriceps endurance	
	*r*	*p* value
Sitting- JPS in 20° of flexion	-0.48	< 0.001
Sitting- JPS in 40° of flexion	-0.62	< 0.001
Sitting- JPS in 60° of flexion	-0.58	< 0.001
Standing- JPS in 20° of flexion	-0.25	0.084

*JPS* Joint position sense, *KOA* knee osteoarthritis. The correlation was tested using Pearson's correlation coefficient analysis

There were statistically significant differences in quadriceps endurance holding times (d = 7.09, p0.001) between asymptomatic and KOA subjects when analyzed using independent t-tests (Table 3). Compared to the asymptomatic group, the KOA group's quadriceps endurance holding time was significantly lower. The magnitude of proprioceptive JPS were larger in individuals with KOA compared to asymptomatic groups in sitting positions (20° of flexion: d = 1.73, *p* < 0.001; 40° of flexion: d = 1.72, *p* < 0.001; 60° of flexion: d = 1.83, *p* < 0.001), and standing position (20° of flexion: d = 1.89, *p* < 0.001). (Table 3).

**Table 3:** Between the group's comparison of the quadriceps endurance and knee JPS in unilateral KOA (*n* = 50) and asymptomatic (*n* = 50)

Variable	KOA individuals (*n* = 50)	Asymptomatic (*n* = 50)	Mean difference (95% CI)	p	Cohen's d
Quadriceps Endurance (sec)	56.38 ± 16.49	150.44 ± 8.92	94.06 (88.80, 99.32)	< 0.001	7.09
Sitting- JPS—20° of flexion	5.88 ± 1. 57	3.56 ± 1.05	-2.32 (-2.85, -1.79)	< 0.001	1.73
Sitting- JPS—40° of flexion	5.70 ± 1.49	3.60 ± 0.86	-2.10 (-2.58, -1.62)	< 0.001	1.72
Sitting- JPS—60° of flexion	5.94 ± 1.71	3.44 ± 0.88	-2.50 (-3.04, -1.96)	< 0.001	1.83
Standing- JPS—20° of flexion	5.28 ± 1.26	3.02 ± 1.12	-2.26 (-2.73, -1.79)	< 0.001	1.89

*KOA* Knee osteoarthritis, *JPS* Joint position sense, *CI* Confidence Interval

## Discussion

This research aimed to see a relationship between quadriceps endurance and knee joint JPS in individuals with unilateral KOA and compare the quadriceps endurance holding time and knee JPS between KOA and asymptomatic individuals. This research adds a vital component to the findings of previous studies on people with persistent KOA. The quadriceps endurance showed a negative correlation with knee JPS, and the subjects with KOA showed significantly lower quadriceps endurance holding capacity and larger JPS magnitude compared to asymptomatic.

To our knowledge, there is little research on the relationship between quadriceps isometric endurance and knee proprioception in people with unilateral KOA. In a recent study, Kim et al. found no significant relationship between knee extensor muscle strength and proprioception in 40 females with KOA aged 65 to 75 years [20]. Our study showed a significant negative correlation between quadriceps endurance and proprioception errors. However, these results cannot be compared with Kim et al.'s study, as methodological differences exist between the two studies. Our research focused on the relation between endurance and proprioception, while Kim et al. assessed the association between strength and proprioception [20]. In addition, the average age of individuals with KOA was greater in this study (67.1 ± 4.36), suggesting that older people with persistent knee pain may be less resilient to pain intensity and muscle endurance [26]. As a result, the study's significant association between knee extensor endurance and proprioception might be explained. The quadriceps muscles are thought to be responsible for maintaining posture, which is reflected in the endurance holding capacity [29]. We predicted the two activities to be linked because the same group of muscles has been proposed to be rich in muscle spindles that send proprioceptive afferents to the CNS, and the hypothesis was approved.

Robust literature is available on the association between muscular endurance and proprioception in different chronic conditions and pathologies other than the knee joint. Reddy et al. [27] discovered a significant association between proprioceptive sensibility and neck extensor endurance in chronic neck pain patients [30]. Hu et al. [31], in empirical research., demonstrated that more significant joint position errors were associated with lower muscle endurance and vice versa chronic muscle and joint dysfunction subjects. The current investigation findings were consistent with those of Miura et al. [32]. In 27 healthy male volunteers, he found a relationship between muscular fatigue and proprioceptive impairment. Muscle fatigue is thought to have a negative impact on proprioception due to a lack of activation in mechanoreceptors [30, 33–38]. Knee extensor muscle dysfunction may impair proprioceptive afferent signals to the higher centers, thereby activating irregular motor patterns [39]. In individuals with reduced quadriceps endurance, muscle activation patterns may be inhibited, affecting knee movement patterns and deteriorating position sense [40].

The findings of this study are comparable with earlier studies that found similar results when comparing quadriceps endurance holding capacity between persons with and without KOA [20, 41]. Lee et al. [42] and Alnahdi et al. [43] reported KOA individuals with reduced quadriceps strength and endurance [42, 43]. Individuals with KOA have been observed to have muscular inhibition and impaired force-generating capabilities, resulting in voluntary inactivation of the quadriceps and hamstrings muscles [44, 45]. Compared to age- and sex-matched controls, subjects with KOA are functionally limited [46]. Because quadriceps endurance may be a significant determinant of physical function in people with KOA, these limits could be attributable to quadriceps muscle weakness. Some people argue that because pain intensity and knee pain-related functional impairments are linked to quadriceps endurance, the two factors should be included in the definition of quadriceps endurance [4]. It should be highlighted that all these conclusions are based on the clinical quadriceps endurance test for the knee extensor muscle group.

In addition, in this study, individuals with KOA had reduced proprioceptive responsiveness. Regardless of its direction, the absolute error measures the extent of the repositioning inaccuracy. In previous investigations, individuals with KOA had more absolute errors in all the tested directions than asymptomatic volunteers. Different authors demonstrated reduced proprioceptive accuracy in the knee with increased pain and disability. These changes will compromise knee stability and cause degenerative changes to damage the knee joint [47].

In literature, different authors have suggested sitting or standing positions to evaluate knee JPS [48–50]. The sitting position is considered a more stable, while the standing position is considered more functional [49, 50]. The previous results are conflicting, and there is no best position for measuring knee JPS [48–50]. In this study, we assessed knee JPS in standing and sitting positions, the JPS errors in the standing position were smaller compared to sitting. Our results are in accordance with Stillman et al. [48] findings in which he demonstrated smaller knee JPS errors in weight-bearing (standing) or non-weight bearing (sitting) positions. Weightbearing may augment the afferent discharge from compressed mechanoreceptors embedded in the connective tissue structures of WB joints. Standing position is recommended for assessing knee JPS because it involves all muscular, cutaneous, and articular mechanoreceptors to produce efficient proprioceptive acuity.

### Limitations

Quadriceps isometric endurance holding time was recorded when the individuals failed to maintain the leg in the horizontal position, regardless of what caused them to stop the test; in the KOA group, pain may be a substantial component in disease development, highlighting the ability of the quadriceps muscles to generate tension. In addition, muscle endurance may play a role in developing KOA, emphasizing the quadriceps muscles' ability to generate force. This study comprised unilateral KOA diagnoses with increased knee pain and disability. This study results are limited to this population only, and the generalizability of this study results to other knee conditions requires caution.

## Conclusion

This study's results demonstrated a significant negative correlation between quadriceps isometric endurance and knee JPS in unilateral KOA individuals. Furthermore, the quadriceps endurance capacity is lower in people with unilateral KOA, and knee JPS magnitude was larger than asymptomatic individuals. The knee JPS errors were smaller when evaluated in a standing position compared to a sitting position. Standing position may be considered while assessing knee JPS as it is more a functional position. However, a future study with larger sample size is suggested to determine the optimal position for assessing knee JPS. These data imply that proprioception and endurance in people with KOA are two different and independent aspects of knee extensor muscle performance. Thus, improving quadriceps endurance and proprioception may be suggested in managing individuals with KOA.

## Data Availability

On request to the corresponding author Ravi Shankar Reddy (rshankar@kku.edu.sa), all data are available at the Department of Medical Rehabilitation Sciences.

## Abbreviations

KOA: Knee osteoarthritis
JPS: Joint position sense
VAS: Visual analog scale
WOMAC: Western Ontario and McMaster Universities Osteoarthritis Index
